# X-Ray Nanoscopy of a Bulk Heterojunction

**DOI:** 10.1371/journal.pone.0158345

**Published:** 2016-07-01

**Authors:** Nilesh Patil, Eirik Torbjørn Bakken Skjønsfjell, Niko Van den Brande, Elvia Anabela Chavez Panduro, Raf Claessens, Manuel Guizar-Sicairos, Bruno Van Mele, Dag Werner Breiby

**Affiliations:** 1 Department of Physics, Norwegian University of Science and Technology (NTNU), 7491, Trondheim, Norway; 2 Physical Chemistry and Polymer Science (FYSC), Vrije Universiteit Brussel, 1050, Brussels, Belgium; 3 Paul Scherrer Institut, 5232, Villigen PSI, Switzerland; 4 Department of Micro- and Nanosystem Technology (IMST), University College of Southeast Norway, Campus Vestfold, 3184, Borre, Norway; Oregon State University, UNITED STATES

## Abstract

Optimizing the morphology of bulk heterojunctions is known to significantly improve the photovoltaic performance of organic solar cells, but available quantitative imaging techniques are few and have severe limitations. We demonstrate X-ray ptychographic coherent diffractive imaging applied to all-organic blends. Specifically, the phase-separated morphology in bulk heterojunction photoactive layers for organic solar cells, prepared from a 50:50 blend of poly(3-hexylthiophene) (P3HT) and phenyl-C61-butyric acid methyl ester (PCBM) and thermally treated for different annealing times is imaged to high resolution. Moreover, using a fast-scanning calorimetry chip setup, the nano-morphological changes caused by repeated thermal annealing applied to the same sample could be monitored. X-ray ptychography resolves to better than 100 nm the phase-segregated domains of electron donor and electron acceptor materials over a large field of view within the active layers. The quantitative phase contrast images further allow us to estimate the local volume fraction of PCBM across the photovoltaically active layers. The volume fraction gradient for different regions provides insight on the PCBM diffusion across the depletion zone surrounding PCBM aggregates. Phase contrast X-ray microscopy is under rapid development, and the results presented here are promising for future studies of organic-organic blends, also under *in situ* conditions, *e*.*g*., for monitoring the structural stability during UV-Vis irradiation.

## Introduction

Polymer solar cells offer a potential solution to the global energy crisis due to their cost-effectiveness, flexibility, lightweight, large-scale manufacturing characteristics, and efficient conversion of sunlight to electricity [1]. Solar cells based on conjugated polymers acting as electron donor materials blended with fullerene-based electron acceptor material have achieved up to 11.7% power conversion efficiency (PCE) using a single-layer bulk heterojunction (BHJ) device structure [2]. Blends of poly(3-hexylthiophene) (P3HT) and phenyl-C61-butyric acid methyl ester (PCBM) are a benchmark class of photovoltaically active materials [3], forming two partially miscible phases that are segregated in a random fashion. Consequently, a donor and acceptor percolating network is formed, yielding a large interfacial BHJ promoting charge separation.

Imaging of the BHJ active layer morphology has been reported with several techniques, crucially assisting the understanding of its optoelectronic properties. Perhaps most notably, synchrotron-based scanning transmission X-ray microscopy (STXM) in combination with near-edge X-ray absorption fine structure (NEXAFS) has been used to probe the electronic structure of fullerene materials and derivatives [4]. The morphology of nanostructured organic thin films by quantitative chemical mapping of the bulk heterojunction composition with lateral high resolution of down to 10 nm has been reported [5–7]. Although STXM has become a routine method for imaging organic film structures, it has several disadvantages. Chiefly, the penetration depth of soft X-rays limits the total sample thickness to < 100 nm [8]. In practice, the film must thus be free-standing (lifted onto a Cu-grid), and *in situ* experiments become practically infeasible.

Optical and electron microscopies [9–10] have been used to show that thermal annealing of the active layer leads to a coarsening of the P3HT-PCBM morphology and, over extended periods of time, the formation of crystalline PCBM domains with micron-sized dimensions [11–12]. With the enhanced molecular mobility obtained during thermal annealing, the PCBM molecules diffuse within the layer to form aggregates or crystals [12]. Atomic force microscopy (AFM) and scanning tunneling microscopy (STM) have been used to analyze the local functionality and structural heterogeneities in organic thin films [13–15]. Recent AFM studies suggest that longer side-chain polyalkylthiophenes enable higher diffusion rates of PCBM in the polymer, leading to large-scale phase segregation, significantly reduced interfacial area and thus less photocurrent generation [16].

Using transmission electron tomography, 3D images of the morphological organization of a BHJ active layer have also been reported [17]. Distinctively, the domain size distribution and the tortuosity of charge transport paths have been quantified in terms of a three-phase morphology [18]. To ensure further improvements of photovoltaic performance, quantitative structural characterization including the spatially resolved electron density *ρ*(**r**), giving access to the distribution and size of the phase-segregated domains at multiple length scales, is a prerequisite. In addition to usually yielding qualitative images in the sense that intensity variations are hard to interpret, electron microscopy requires high vacuum that can modify the fragile samples, and the electron beam itself is highly damaging to soft materials.

**Fig 1:**
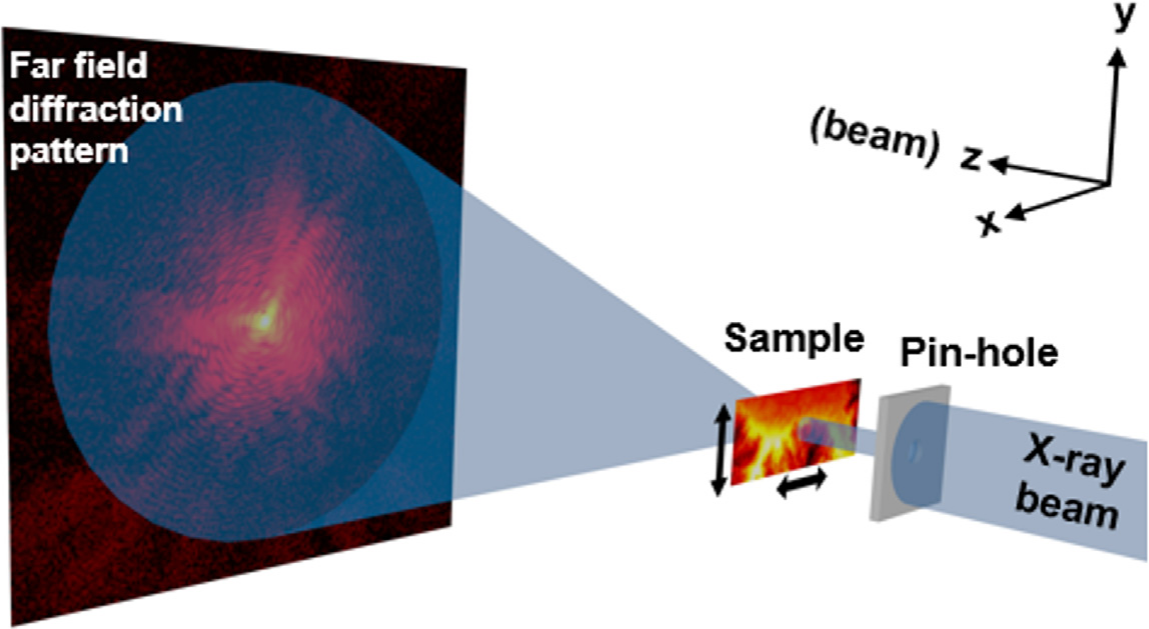
Sketch of the experimental setup for transmission X-ray ptychography. Coherent diffraction patterns are recorded by a 2D detector for a set of partially overlapping scanning positions, allowing numerical reconstruction of the projected complex-valued image of the sample.

Several novel X-ray microscopy methods with high relevance to organic solar cells have recently been developed, yielding improved contrast and resolution through coherent diffractive imaging (CDI) [19] both with soft [20–21] and hard X-rays [22–23], or resonant scattering of polarized soft X-rays [24]. In particular, scanning-CDI, also known as ptychography, *cf*. Fig 1, has emerged as a valuable technique to provide quantitative phase-contrast imaging and high spatial resolution [21, 25–26, 27]. The concept of ptychography is to obtain real space images by applying iterative phase retrieval algorithms [28–29] to a series of diffraction patterns. The sample is laterally shifted with respect to the incoming beam (the “probe”) while maintaining a significant spatial overlap between neighboring diffraction exposures, and one diffraction pattern is collected at each point. The overlap is used as a constraint during the numerical phase retrieval (image reconstruction). In essence, ptychography retrieves the complex-valued transmission of the sample, that is, both phase and amplitude, and it requires neither high absorption nor a strong phase shift to give detailed and reliable images. The resulting image resolution is in principle limited only by the numerical aperture (given by the wavelength and the highest accessible scattering angle), but in practice also the measurement signal-to-noise ratio and mechanical instabilities [25] become important. The appealing aspects of X-ray ptychography include that it yields high-resolution quantitative images over a large field of view, with a modest radiation dose, and that it allows *in situ* microscopy experiments with complex sample environments [30–32].

## Results and Discussion

**Fig 2:**
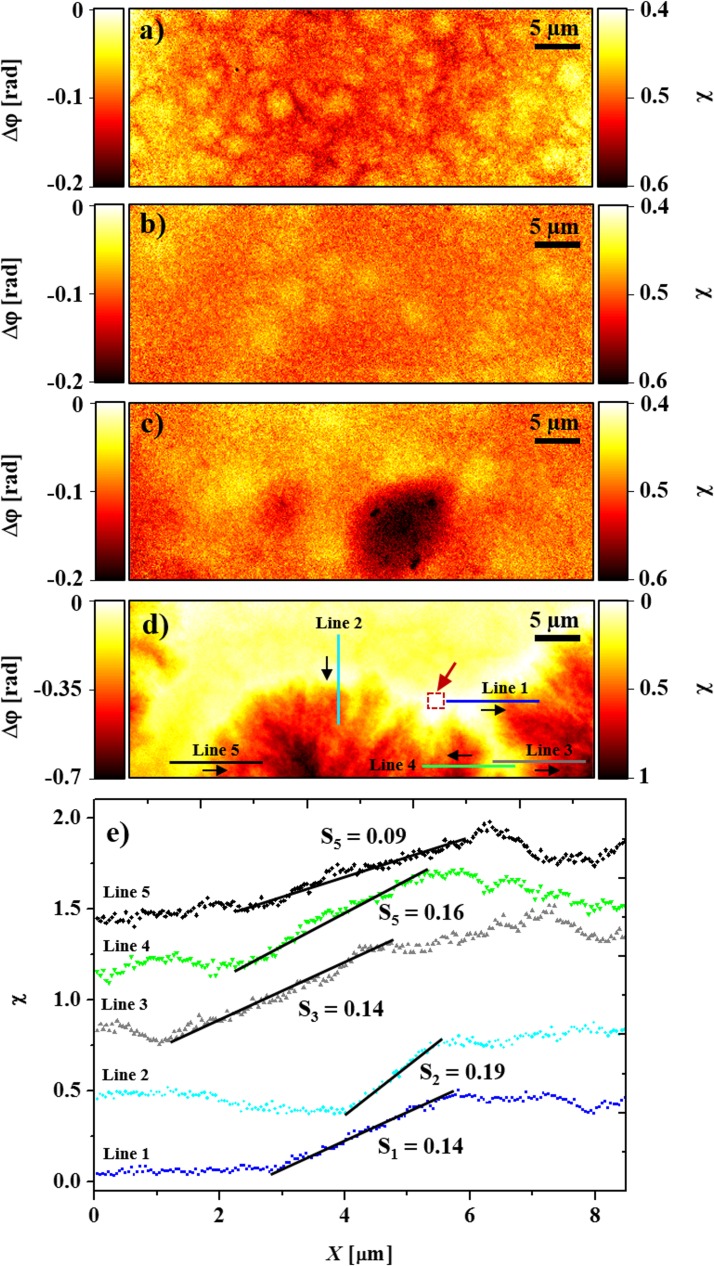
Reconstructed phase contrast high-resolution projections of P3HT/PCBM layers, all measured at room temperature. a) Non-isothermally annealed; b-d) Isothermally annealed with increasing annealing time of 60 s, 720 s and 7500 s at 127°C. e) Estimated volume fraction (χ) as a function of position estimated along the five lines indicated in d). The volume fraction gradient for the five different regions is in the range of 0.14 ± 0.05 μm^-1^. For readability, the curves are vertically offset. Note that the intensity scale differs between a-c) and d), as the aggregation was much more pronounced in the latter case. The red arrow in (d) indicates an area assumed to contain the lowest PCBM concentration of 4%.

The reconstructed phase-contrast projections at room temperature for four active-layer samples subjected to different thermal annealing protocols (see Methods section) are shown in Fig 2. Gratifyingly, the domains of electron donor and electron acceptor materials are clearly resolved. Because the films are sufficiently thin, the fact that the images are projections of truly three-dimensional structures only marginally affect the interpretation; in other words, judged by the images, the films are likely to be of single domain thickness. The phase-contrast images further allow us to estimate the relative volume fraction of the two materials across the area of active layer under investigation. Note that the absorption images (not shown) exhibit essentially no features–these films are effectively phase objects at 6.2 keV photon energy.

The four samples exposed to different thermal treatments show large morphological differences, *cf*. Fig 2A–2D. The mean feature size differs with respect to thermal annealing treatment where the increase in the size of features from a few hundred nanometer to tens of micrometer is observed with the increase in annealing time (refer Fig 2C and 2D). On the basis of the electron density differences (*ρ*_e(PCBM)_ ≈ 0.7 Å^-3^ and *ρ*_e(P3HT)_ ≈ 0.4 Å^-3^) between PCBM and P3HT reported in the literature [33], the darker regions are PCBM-rich domains, while the lighter regions are P3HT-rich. In particular, the sample annealed for 7500 s shows large domains, and for further analysis, we assumed that the regions in the 7500 s sample showing the largest (smallest) phase shifts correspond to essentially pure phases of PCBM (P3HT) [34]. By further making the admittedly crude assumption that the films were all of equal and uniform thickness, in spite of the facts that PCBM is known to aggregate also vertically [35], and that the phase shift can also be a result of variations in other parameters such as nanoporosity and composition, we could then estimate the relative volume ratio of PCBM by linear interpolation.

The low contrast of the features in the P3HT/PCBM active layer subjected to cooling directly from the melt to room temperature (“non-isothermal annealing”, see Methods section), *cf*. Fig 2A, indicates an evenly mixed composition of small and weakly developed domains. It is interesting to note that phase separation is clearly present, indicating that the used cooling rate of 20°C min^-1^ from the melt is insufficient to prevent phase separation. This further emphasize that the morphology of an annealed active layer is not solely caused by the isothermal part of the annealing treatment. With increased thermal annealing more pronounced morphological inhomogeneities develop, and for the longest annealing time, the active layer was found to exhibit a pronounced phase segregation of P3HT and PCBM. The 7500 s sample has domains resembling spherulitic growth of crystals, with fractal-like structures as seen in diffusion-limited aggregation [36]. Such large domains are not suitable for the achievement of higher power-conversion efficiency solar cells because of the reduced interfacial area between the donor and acceptor domains, however the merit of the experiments reported here is to demonstrate that these domains can indeed be studied using X-ray ptychography.

Fig 2E shows estimated volume fraction profiles (PCBM concentration gradient) extracted from the sample subjected to isothermal annealing for 7500 s. The volume fraction gradient varies in a narrow interval from 0.09 μm^-1^ to 0.19 μm^-1^. This indicates that the dynamics of the PCBM diffusion out of the depletion zone is similar across the sample. A rough estimate of the diffusion coefficient, yielding *D* = 7 x 10^−12^ cm^2^ s^-1^ was performed using analysis similar to that reported by Dastoor *et al* [8, 37], see short discussion on diffusion analysis in the Methods section.

**Fig 3:**
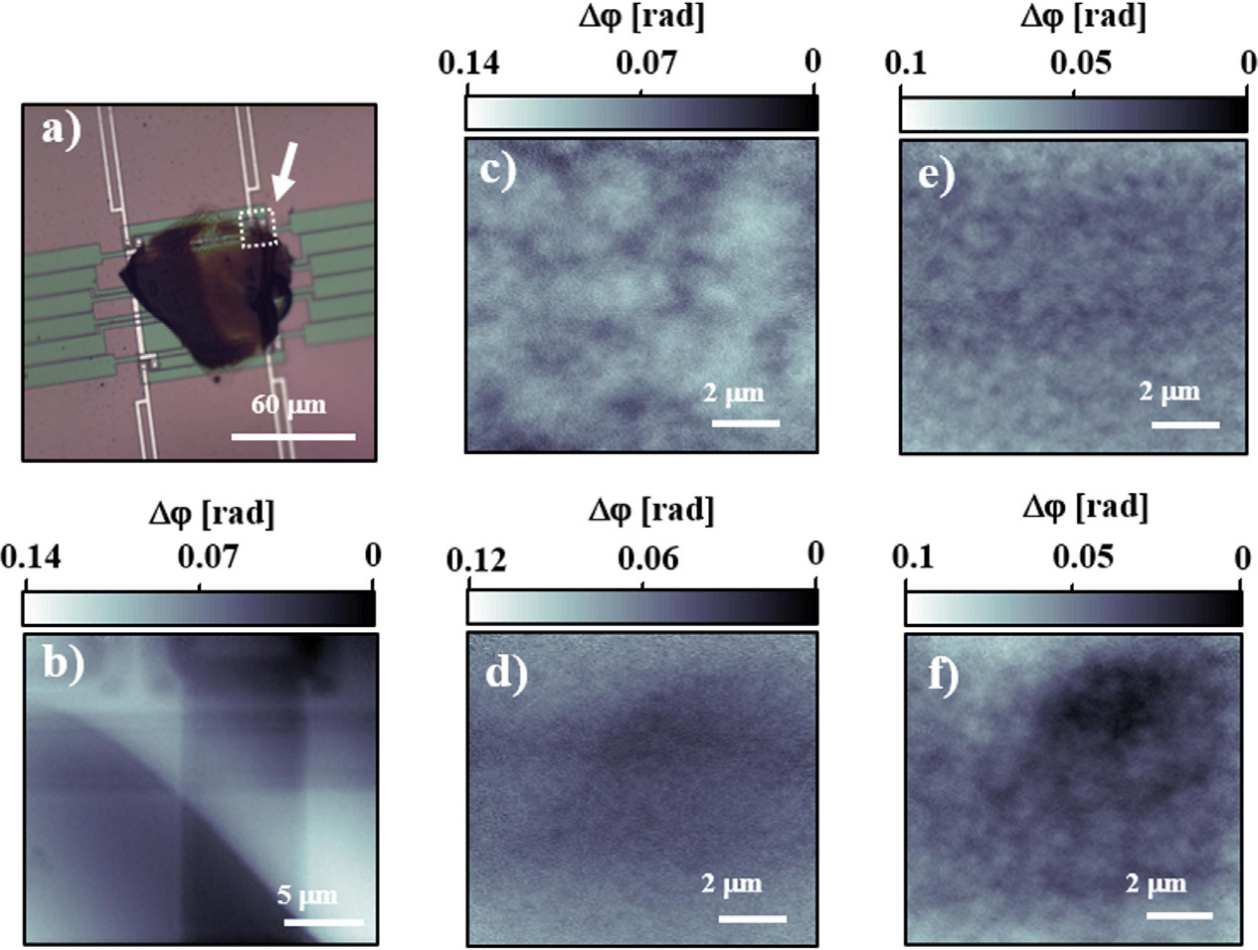
Results obtained with fast-scanning calorimetry sample holder. a) Optical micrograph of the active area on the electronic chip. The central patch is the P3HT-PCBM sample. b-f) Reconstructed phase-contrast high-resolution ptychography projections, with (b) showing the area corresponding to the section marked by the outlined white square in (a). c) Projection for as-cast film, showing that the deposition process has induced a certain morphology. d) Morphology after erasing the thermal history by shortly visiting the melt, showing an essentially featureless image. e),f) Images obtained after 60 s and an additional 660 s (total of 720 s), respectively, of annealing at 400 K, clearly showing that a coarser morphology develops with time. All images were collected at room temperature to reduce problems with radiation damage.

In order to better understand the morphology and to further investigate the possibilities of future *in situ* experiments, a custom sample cell featuring a fast-scanning calorimeter chip [38–39], was designed and installed at the beamline. This sample cell allowed ultrafast heating and cooling of the miniature film. A result of considerable interest, is the observation that <5 s of residence at *T* = 550 K (which is above the melting temperature of PCBM), removes the thermal history completely, as judged from the essentially featureless phase-contrast image. Convincingly, subsequent image captures after repeated increasingly long annealing times at *T* = 400 K, showed clearly a non-uniform morphological structure gradually developing from small scale, see Fig 3. To reduce problems of radiation damage, all these captures were made at room temperature (between the annealing steps).

The reported spatial resolution of about 50–100 nm is too poor to resolve the relevant length scale of exciton diffusion [40]. However, the promise of coherent diffractive imaging is precisely that the needed resolution of about 10 nm is currently becoming within reach [21, 25–26, 41], suggesting that this method will become highly important for the continued development of organic solar cells, much like grazing incidence X-ray scattering methods have become indispensable for resolving thin film structures at the molecular scale [42–45]. Natural next steps will be *in situ* studies of domain dynamics and degradation [46], and also 3D investigations through tomographic methods that will lift the constant-thickness assumption and thereby enable detailed studies of the phase interfaces.

For ultimate photovoltaic performance, having a bicontinuous morphology with a characteristic length scale of the domains in the range of 10–15 nm, comparable to the exciton diffusion length, is essential. The pioneering experiments presented here provide a reconstructed pixel size of 45 nm, with the obtained spatial resolution closer to 100 nm. Therefore, the characteristic length scale (> 100 nm) of the observed domains reported here would not be suitable for the appropriate functioning of a real device. The large-size domains are a consequence of extended-time thermal annealing procedures, and can in principle mimic long-term solar cell degradation. To optimize the nanomorphology in the length scale of ≤ 100 nm for actual solar cell applications, other annealing schemes must be employed [38–39]. Nevertheless, the observation of the large domains in the present study is another step forward in using hard X-ray ptychography as a future characterization tool for a wide range of organic thin films. The organic nature of the samples, with relatively low radiation tolerance and weak scattering, effectively limits the achievable resolution, but with more sophisticated sample environments [20, 25], possibly including cryogenic protection, a significantly better resolution in the near future is expected.

An important point to note is the challenges caused by X-ray radiation damage which restricts better resolution in the imaging of organic materials. Radiation damage was observed, in particular if attempting to expose the sample at elevated temperatures. At long exposure times, the beam significantly damaged the morphology within the active layers (data not shown). One way of evading this problem in the future might be to use cryogenic temperatures, but this will clearly preclude *in situ* studies.

## Conclusion

In conclusion, we have introduced X-ray ptychographic nanoscopy as a tool for characterizing organic-organic blends, exemplified with a study of the morphological features in P3HT/PCBM active layers subjected to different thermal annealing treatments. The phase-segregated domains of P3HT and PCBM were clearly resolved within the reconstructed high-resolution phase-contrast projections and allowed us to estimate the relative volume fraction of PCBM across the active layer. Using a custom sample cell featuring fast-scanning chip calorimetry, we could also follow the morphological developments as function of annealing time. The reconstructed high resolution images of phase segregated domains of electron donor and electron acceptor materials in the P3HT/PCBM active layer demonstrate that X-ray ptychography already is a powerful technique for imaging low contrast nanostructured organic materials, and with future experimental improvements, routine *in situ* quantitative imaging can be expected.

## Methods

### Fabrication of active layer and application of thermal annealing treatments

Electron donor material P3HT (*M* ~ 10^5^ g/mol) and electron acceptor PCBM (*M* ~ 910.9 g/mol) were purchased from Rieke Metals Inc. and Solenne BV, respectively, and used as received. Solutions of P3HT/PCBM (1:1) mixtures (by weight) were prepared by dissolving a mass of P3HT and PCBM to chlorobenzene, yielding a 5 wt% solution. P3HT/PCBM mixture was spin-coated at 100 rpm for 60 s onto X-ray transparent silicon nitride membrane (*A* = 1.5 mm x 1.5 mm) to obtain the BHJ active layer with a thickness of ~1 μm. Several active layer samples were fabricated and allowed to dry under atmospheric conditions.

The active layers were subjected to both isothermal and non-isothermal annealing schemes using differential scanning calorimetry (DSC). The experimental thermal history for isothermal annealing is summarized as follows. The sample was i) heated from room temperature to 280°C to melt; ii) held at 280°C in the melt for 2 min to remove any initial structures; iii) cooled to the isothermal temperature of 127°C; iv) kept at isothermal temperature for different times: 60 s, 720 s and 7500 s; v) cooled to 20°C. For non-isothermal annealing, the sample was heated from 20°C to 280°C and held at 280°C for 2 min to remove initial structures, followed by cooling to 20°C. All heating and cooling steps throughout the thermal annealing treatments were performed with a controlled rate of 20°C min^-1^.

In a separate series of experiments with a custom made sample cell featuring a fast-scanning calorimeter chip (details to be published elsewhere), the miniature thin film sample (coated onto a 60 x 60 μm^2^ silicon nitride membrane) could be heated to melting and cooled back to room temperature with rates of ~10^4^ K s^-1^. This setup allowed the effects of repeated annealing of the same sample to be imaged at room temperature.

### X-ray ptychography measurements

The X-ray ptychography experiment was performed at the cSAXS beamline at the Swiss Light Source (X12SA, Paul Scherrer Institut, Villigen, Switzerland) with 6.20 keV photon energy, corresponding to an X-ray wavelength of *λ* = 1.99 Å, selected with a double-crystal Si(111) fixed-exit monochromator. The distance of 7.45 m between the sample and the detector was maintained for all measurements, and a helium-filled flight tube was positioned between the sample and detector in order to reduce absorption and scattering by air. The experimental setup is illustrated schematically in Fig 1. A coherent patch of the X-ray beam passed through a 3 μm pinhole, located 4 mm in front of the sample. A custom-made sample holder was fitted onto a XYZ nPoint piezoelectric nanopositioning stage. The sample was scanned laterally across the beam using the piezoelectric stage in a Fermat spiral pattern [47] with an average step size of 1 μm and counting time of 0.5 s for each of the 100 exposures per scan. The diffraction patterns were recorded with a pixelated hybrid Pilatus 2M detector [48] with 172 μm x 172 μm pixel size. Each scan covered a field of view of 10 μm x 10 μm, and 3 x 9 such scans were stitched together with an overlapping field of view of 5 μm between each scan, by reconstructing a common object for several scans following the procedure described elsewhere [49]. In this manner we could form an image with a large effective field of view with 20 μm x 50 μm while minimizing the effects of a slowly changing illumination by reconstructing a separate probe for each individual scan. For these measurements, an average incident flux of 1.46 x 10^7^ photons/μm^2^ reached the sample with a corresponding absorbed radiation dose estimated to 77 kGy for P3HT and 21 kGy for PCBM, in average 49 kGy for the P3HT/PCBM blend.

### Ptychography reconstruction and analysis

X-ray phase contrast imaging is based on the visualization of changes in the wave front when the X-ray beam passes through the sample. The interaction of X-rays with materials is described using a complex-valued refractive index [50],

$$n=1-\delta +i\beta =1-\left(\frac{N\lambda^{2}r_{e}}{2\pi }\right)+i\beta$$ (1)

where the decrement *δ* is associated with the phase shift of the transmitted wave, and the imaginary part i*β* is associated with absorption. *r*_e_ is the classical electron radius, *N* the number of electrons per unit volume, and *λ* the X-ray wavelength. The magnitude of the imaginary part in (1) is related to the linear absorption coefficient *μ*_0_ through *β = λμ*_*0*_*/(*4*π)*. The phase shift of X-rays propagated through a sample relative to vacuum is for a thin sample approximated by

$$\Delta \phi (x,y)=\frac{-2\pi }{\lambda }\int \delta (x,y,z)\text{dz}\approx \frac{-2\pi }{\lambda }\delta (x,y,z)d$$ (2)

The integration is carried out over the path of X-ray beam through the sample, and the X-ray propagation direction is taken to be in the *z*-direction. The latter approximation in (2) holds when the film sample is uniform over the thickness *d*.

In ptychography, redundancy, which allows phase reconstruction, is introduced by multiple measurements with partially overlapping illuminated regions on the sample. More specifically, at each scanning position *j*, the complex valued two-dimensional exit wave at the sample corresponding to the *j*^th^ diffraction pattern can be described as *ψ*_j_ (**r**) = P(**r**—**r**_j_)*·* O(**r**), where P(**r—r**_j_) is the “probe” wave field and O(**r**) is the sample transmission function with **r** ≡ (*x*, *y*) being the position vector. The intensity distribution *I*_j_, measured in the far field, can be expressed as *I*_j_(**q**) = ${\left|\tilde{\psi_{\text{J}}}\;(q)\right|}^{2}$, where $\tilde{\psi_{\text{J}}}(q)$ is the Fourier transform of *ψ*_j_(**r**), and **q** ≡ **k**_f_ *-***k**_i_ is the scattering vector with **k**_i_ and **k**_f_ being the wavevectors of the incoming and scattered beams, respectively. Provided that there is sufficient overlap between neighboring illuminated regions of the sample, both P(**r**) and O(**r**) can be reconstructed simultaneously from the intensity measurements [23, 51]. For the ptychographic reconstructions, a nonlinear optimization algorithm [29, 51] with a maximum of 300 iterations was used, using the central 192 x 192 detector pixels of the collected diffraction patterns. Linear phase terms, which are inherent degrees of freedom for ptychography when the probe is simultaneously retrieved, were removed by considering a measured region outside the sample [52].

In this article, the samples studied were too weakly absorbing to give recognizable information in the absorption contrast signal (data not shown). Also the phase shift observed, |Δ*ϕ*|, was in all cases < π/4, and has been shifted for all samples such that a phase shift of zero corresponds to the mean value within regions of assumedly lowest PCBM concentration of 4% [53] (as exemplified by the marked area in Fig 2D)–any additional phase shift is thus to a first approximation due to the increasing concentration of PCBM. For linear interpolation of Δ*ϕ* to χ, the largest measured phase difference which was obtained in the active layer with 7500 s of isothermal annealing treatment was considered. The formula used for linear mapping of Δ*ϕ* onto χ⊰ (0, 1) is given by

$$\chi =\frac{\Delta \phi -\phi_{\min}}{\phi_{\max}-\phi_{\min}}$$ (3)

Here *ϕ*_min_ and *ϕ*_max_ are the minimum and maximum value of the phase shift, respectively. For the other images a)-c), an additional assumption to handle the phase offset was that the average composition χ should be close to 0.5.

### Diffusion analysis

The diffusion of PCBM in the P3HT phase governs the formation of a co-continuous structure including both crystalline and amorphous phases of P3HT within the active layer. The phase segregated PCBM domains is perceived to influence the ordering of the P3HT phase and efficient device performance. The diffusion coefficient of PCBM is an important subject and a range of values have been reported in recent years for different annealing temperatures, for instance, i) 2.5 x 10^−10^ cm^2^ s^-1^ for 140°C [8], ii) 5 x 10^−14^ cm^2^ s^-1^ for 130°C [54], and 1.1 x 10^−14^ cm^2^ s^-1^ for 160°C [55], iii) 2.2 x 10^−11^ cm^2^ s^-1^ for 50°C and 5.7 x 10^−11^ cm^2^ s^-1^ for 70°C [56], respectively. The diffusion rate of PCBM in the P3HT phase can be determined from the 1D composition profile using Fick’s second law of diffusion and assuming an initially even distribution of PCBM. Fick’s second law of diffusion is given by

$$\frac{\partial \chi }{\partial t}=-D\frac{\partial^{2}\chi }{\partial^{2}x}$$ (4)

Here χ is the PCBM concentration, *D* is the diffusion coefficient, *x* and *t* denote position and time. The PCBM diffusion coefficient can be obtained by fitting the PCBM volume profile to a one-dimensional solution to Fick’s second law as demonstrated by Dastoor *et al* [8, 37]. Under the assumptions of an initially uniform concentration of PCBM, and effective sinks at *x* = 0 and *x* = *L*, standard Fourier analysis gives the equation

$$\chi (x,t)=\chi_{\text{b}}+(\chi_{0}-\chi_{\text{b}})\frac{4}{\pi }\sum_{m=1}^{\infty }\frac{1}{\left(2m-1\right)}\sin\left(\frac{(2m-1)\pi x}{L}\right)\exp\left[-Dt{\left(\frac{(2m-1)\pi }{L}\right)}^{2}\right]$$ (5)

**Fig 4:**
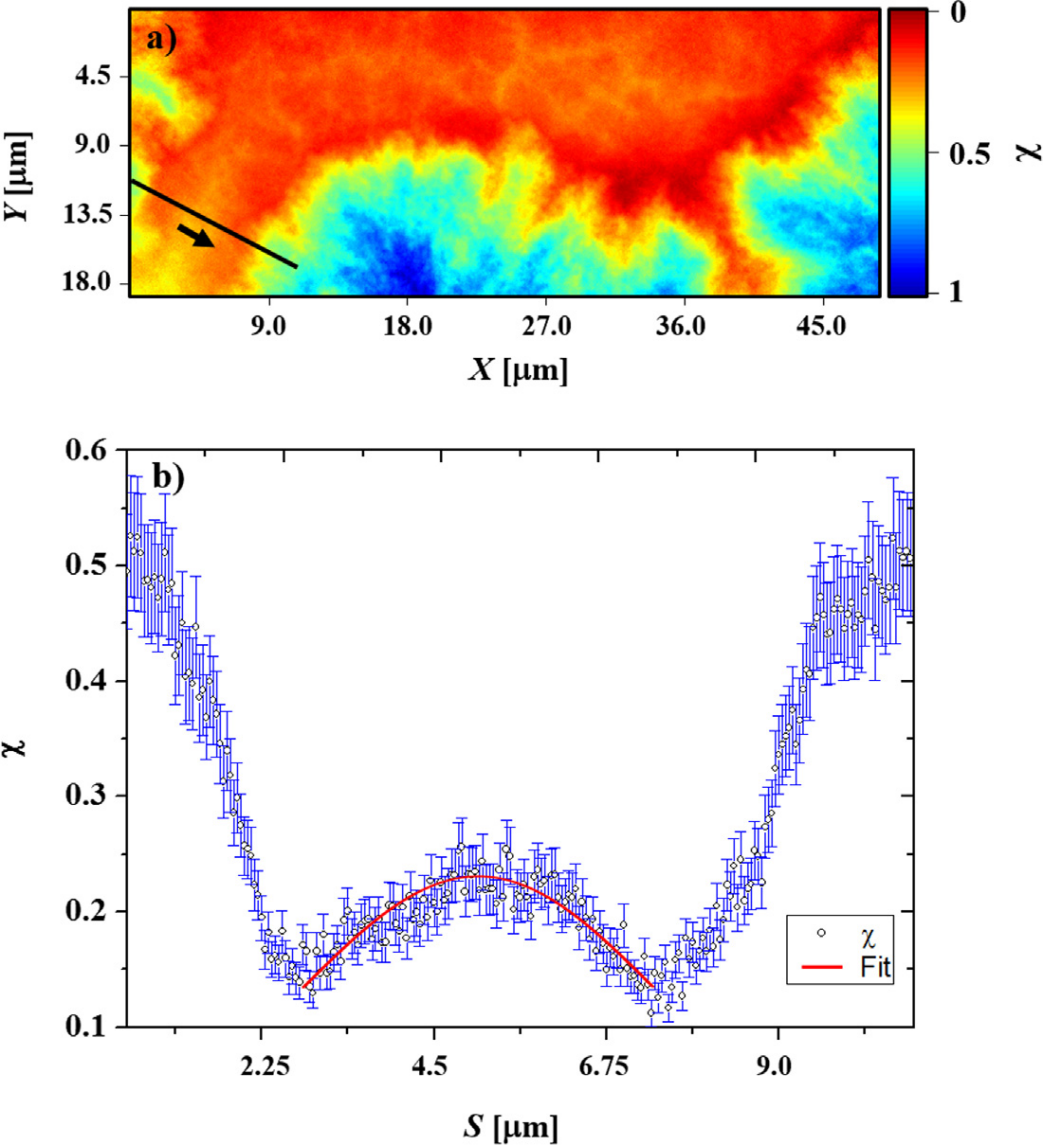
(a) Reconstructed phase contrast high resolution projection of P3HT/PCBM layer isothermally annealed for 7500 s at 127°C. The black line in the projection image corresponds to the PCBM concentration profile shown in (b). The red curve in (b) shows the fitting analysis applied to the PCBM concentration profile using Eq 5. The fitted parameters of *L* = 6.0 μm and *D* = 7 x 10^−12^ cm^2^ s^-1^ provide excellent agreement between the experimental data and the model.

Here *L* is the distance between neighboring PCBM aggregates, χ_0_ = 50% is the initial concentration of PCBM in the active layer and χ_b_ = 12% is the estimated concentration at the base of the aggregate. The area with the highest concentration of PCBM was assumed to be pure PCBM. An assumption for the diffusion analysis was that the distance between the PCBM aggregates is 6.0 μm (*c*.*f*. Fig 4). The diffusion coefficient was fitted to be 7 x 10^−12^ cm^2^ s^-1^ corroborating earlier studies.
